# 

**Published:** 1885-07

**Authors:** 


### EDITORIAL.

The Twenty-Fifth Annual Meeting of the American Dental Association will be held at Minneapolis, commencing August 4th, 1885. The present prospects are that the meeting will be an unusually large one. The railroad rates have been secured at an unprecedentedly low rate. Tickets for the round trip from New York to Minneapolis and return will be furnished for $24.00. Round trip from New York to Chicago and return for $18.00. Round trip from Chicago to Minneapolis and return $6.00.

At present it will be necessary for those wishing these tickets to secure them in Chicago. Later we may be able to make arrangements by which they can be secured at different points east.

By sending check for tickets to Chairman of Committee of Arrangements the tickets will be promptly forwarded.

Negotiations are pending for rates from other points that the committee anticipate will accomodate all, and more definite information will be given in later journals, and also in a circular sent to every member.

The hotel rates are as follows: West Hotel, $4.00 per day; Nicollet House, $3.00 per day; National Hotel, $2.00 per day. It is expected that a reduction from these will be made.

It is hoped that members having any new facts or ideas in regard to theory or practice will come prepared to present them in connection with the section work. Any one having any thing new in the way of appliances will be given an opportunity to demonstrate their use during the half day that will be devoted to clinics.

### ATTRACTIONS AND EXCURSIONS.

Come equipped with guns and fishing tackle. While the interest and benefit of the meetings the attractions of the trip and the beautiful city where we meet are too well known to need special mention, it may not occur to all that they will find themselves in Minnesota, in one of the finest of hunting and fishing countries. Minnesota is especially famous for its prairie chicken and grouse shooting, and its fine fishing grounds.

It is estimated that there are no less than 10,000 lakes dotting the State.

If one wishes a still greater variety of scenery, to see a new, wild and picturesque country, to draw out the big brook trout, the black bass, the "gamy" pickerel and the mighty muskallonge from the cold waters of the Lake Superior region, in fact to enjoy the finest fresh water fishing in the world, a round trip ticket from Chicago to Ashland and return will be furnished them for $10.00.

A still greater attraction (if one more were needed) is offered in shape of a ten day's excursion to the far-famed " Yellow Stone National Park," immediately upon close of the Association, provided a sufficient number send in their names to warrant the securing of special cars and special rates. The committee believe that when so far on the way as Minneapolis many will wish to avail themselves of this opportunity of seeing the grandest scenery in the world. The entire expense for the round trip from Minneapolis, including rail transportation, Pullman sleeping car fares, meals on Northern Pacific dining cars, hotel accommodations, five days in the park, and stage transportation, will be $120. A circular describing the magnificent scenery in full will be sent to every member of the American Dental Association at an early day. Others than members who may contemplate going will receive the same by making application for it.

Come one, come all and bring your wives along. It will be a trip that ladies will especially enjoy. Those wishing to go to Yellow Stone Park will please send in their names at an early day, that all arrangements may be speedily and satisfactorily completed.

For further information, address J. N. Crouse, 2101 Michigan Avenue, Chairman of Committee of Arrangements.

Since the above arrangements were made, a rate has been fixed from Cincinnati to Chicago, and return at $12, thus making the round trip from Cincinnati to Minneapolis $18, which is very moderate, and is within the reach of almost every one who really desires to attend the meeting of the Association. The trip aside from the meeting is worth far more than its costs. We shall expect to meet a large representation of the profession, especially from the West and Northwest.—[*Editor* REGISTER.]

### Pennsylvania State Dental Society.

The Seventeenth Annual Meeting of the Pennsylvania State Dental Society will convene at Cresson Springs, Pa., Tuesday, July 28th, 1885, at 10 A.M., and continue in session three days. Rates at the Mountain House have been reduced from $4 to $3 per day to delegates and their families, dating from Saturday, July 25th, and continuing as long as desired. Orders for *special* excursion tickets will be issued on all lines of the Penna. and A. V. R. R. Usual excursion rates on other roads. Orders or general information can be obtained by addressing,

W. H. FUNDENBERG,

Corresponding Secretary.

958 Penn avenue, Pittsburg, Pa.

THE fifteenth annual session of the New Jersey State Dental Society will be held at the Coleman House, Asbury Park, Wednesday, July 15th, and continue in session three days. Every effort has been made to make this particular session the most memorable of any heretofore held. Interesting papers have been promised from the most eminent in the profession, and the society membership have also contributed liberally. The clinics to be held will be made an important feature, and considerable time and attention have been given towards an exhibition of new and important surgical and mechanical appliances for use in dentistry. It is in contemplation to give a grand reception to the visiting members of the profession on one evening to be arranged for. The place is easy of access from everywhere. The cuisine the best. $2.50 per day the rate. The location superb, within fifty feet of the surf. The hall for sessions attached to the hotel, commodious and cool.

THE following has been sanctioned by the Kentucky State Dental Society to give definite information to the people upon a single important point.

It is printed upon a small card, to be given to every patient who mày be, in any respect, interested. A proper distribution of these cards would doubtless serve a good purpose:

"All teeth of children cut after four years of age are second or permanent teeth, and if lost, will never be replaced.

These teeth frequently begin to decay shortly after they have come through the gum, hence it is important to have them examined at an early age.

Parents being generally uninstructed on this subject, the State Dental Association has deemed it proper to give them this advice to save their children much suffering and the loss of valuable teeth."

### American Dental Association.

The Twenty-fifth Anuual Session of the American Dental Association will be held at Minneapolis, Minnesota, commencing at 11 A. M. Tuesday, Aug. 4th, 1885.

GEORGE H. CUSHING, *Recording Secretary.*

### National Association of Dental Examiners.

The next regular meeting of the National Association of Dental Examiners will be held in Minneapolis, on Tuesday, August 4th, 1885. A full representation is very much to be desired.

GEORGE H. CUSHING, *Secretary.*

### Hymenial.

MARRIED—June 25th, at the Protestant Episcopal Church, in Ann Arbor, Michigan, Miss Dr. Margaret Humphreys D.D.S., of the University of Michigan, to Mr. Elmar Paine, of Xenia, Ohio.

This will probably involve a change of residence of the Dr., which will be a matter of regret to the host of friends and numerous patients she had in Ann Arbor.

The position she has so acceptably occupied in the Dental College for the past two years will be with difficulty supplied so well, and the change will be regretted by those who are the more interested.

The happy couple have the best wishes of their numerous friends for a joyous and prosperous life voyage. May their cup of joy never be less full than to-day.

### THE DENTAL COLLEGE

OF THE

### University of Michigan.

The Eleventh Annual Session of this Institution will commence on the 1st of October and close on the last Thursday of June, thus making a course of nine months. The regular course of instruction commences at once, and will continue through the term, with the customary vacation.

The students in this department will receive instruction in Anatomy, Physiology, Pathology, Chemistry, Materia Medica, Therapeutics, and Surgery, from the Professors of their respective branches in the *Department of Medicine and Surgery* of the University, where lectures commence, and continue the same as with the Dental College. There will also, in addition, be a special course upon Oral Pathology and Surgery, which will embrace from thirty to thirty-five lectures.

### FACULTY OF THE DENTAL DEPARTMENT.

**J. B. ANGELL, LL.D.** - - - - - - - - - - - - **President.**
**J. TAFT, M.D., D.D.S.** **Principles and Practice of Operative Dentistry.**
**CORYDON L. FORD, M.D., D.D.S.** - - - **Anatomy and Physiology.**
**J. A. WATLING, D.D.S.** - - - **Clinical and Mechanical Dentistry.**
**W. H. DORRANCE, DD.S.** - - - - - - - **Prosthetic Dentistry.**
**J. N. Martin, M.D.,** - - - **Lecturer on Oral Pathology and Surgery.**
**MARGARET HUMPHREYS, D.D.S.** - **Demonstrator of Clinical Dentistry.**
———————————— **Demonstrator of Prosthetic Dentistry.**

Students should be promptly present at the College on Wednesday, September 30th, at 10 o'clock, A.M., to make the preliminary arrangements for entering upon regular work. Seats in the lecture room are assigned by election to students in the order of registration on the Steward's Books; and each student is expected to occupy during the session such seat as he may select. Students, on arriving in Ann Arbor, should call at the Steward's office.

### CONDITIONS OF GRADUATION.

The candidate must be twenty-one years of age. *He must furnish satisfactory evidence of good moral character.*

He must devote three years to the study of his profession. He must attend two full courses of lectures in the Dental College, or one in some college having an equal standard of requirements, and the last one here, and we recommend that he attend three courses regularly.

He must sustain an examination satisfactory to the Faculty in all the branches taught.

A graduate of the Medical College may enter the Senior Class, and, if found qualified, may graduate after two years have been devoted to the study of dentistry.

### FEES AND EXPENSES.

The fees, which must be paid in advance, are as follows:

Residents of Michigan.—Matriculation fee, $10.00; annual dues, $25.00

Non Residents.—Matriculation, $25.00; annual dues, $35.00.

Graduation Fee.—For all alike, $10.00. The admission fee is paid but once, and entitles the student to the privileges of permanent membership in any department of the University. The annual dues are paid the first year and every year thereafter while at the University.

For further particulars, address the Dean of the Dental College, Ann Arbor, Michigan.

**J. TAFT, Dean.**

July-85-6m.

### CONTENTS.

COMMUNICATIONS.

### THE DENTAL REGISTER,

A MONTHLY MAGAZINE DEVOTED TO THE INTERESTS OF THE

**DENTAL PROFESSION.**

*Terms Reduced to* $2.00 *Per Year. Single Copies,* 25 *Cents.*

**EDITED BY PROF. J. TAFT, D.D.S.**

—AND—

**Published by SAM'L A. CROCKER & CO., Ohio Dental and Surgical Depot.**

The volume commences in January and closes in December.

The REGISTER being issued monthly is always fresh, therefore Indispensable to the practitioner who desires to keep posted up with the new inventions and improvements which are daily developed. Neither expense nor effort will be spared to give value and variety to its contents. Articles will be illustrated with well executed engravings whenever they will add to the interest or assist to explanation.

Its extensive circulation and frequency of issue make it one of the BEST DENTAL ADVERTISING MEDIUMS in the United States.

All subscriptions must begin with January or July.

Local or special notices, five lines or less, will be inserted for $3 each insertion; over five lines, 50 cts. per line.

All advertising bills payable in advance, or at furthest, after the issue of the first number containing the advertisement. All transient advertisements to be paid for in advance.

☞CONTRIBUTIONS SOLICITED.

Exclusively Editorial Communications should be addressed to the Editor.

The latest number of the REGISTER may be ordered with or without other goods at any time, and all letters should be addressed to

**SAM'L A. CROCKER & CO., Ohio Dental and Surgical Depot, Cincinnati, O.**

